# Evaluation of the factors limiting biogas production in full-scale processes and increasing the biogas production efficiency

**DOI:** 10.1007/s11356-020-09035-1

**Published:** 2020-05-15

**Authors:** Afamia I. Kouzi, Matti Puranen, Merja H. Kontro

**Affiliations:** 1 Faculty of Biological and Environmental Sciences, Ecosystems and Environment Research Programme, University of Helsinki, Niemenkatu 73, 15140 Lahti, Finland; 2 Labio Ltd, Sapelikatu 7, 15160 Lahti, Finland

**Keywords:** Digested sludge, Post-treatment, Volatile solids, Fixed solids, pH changes, Layer separation

## Abstract

Biogas production from sewage sludge volatile solids (VS) by anaerobic digestion slows down towards the end of the process, among inhibitory factors being pH increase upon ammonia accumulation, poorly digestible biomaterials, and high fixed solid (FS) content. The possibility of concentrating the digested sludge VS (41.7–56.6% on a dry weight basis) by surface and bottom layer separation with biogas post-production was studied. Furthermore, the potential to recycle concentrated VS and digested sludge back to the process after adjusting pH 7.0 to optimal for biogas-producing microbes and after acid, alkali, thermal, and sonolytic treatments was examined. In general, pH 7.0 control alone improved biogas production from the recycled digested sludge the most. An equally good improvement in biogas production was achieved by recycling the digested sludge, which had been heated until ammonia had evaporated and the pH dropped to 7.0 (1–2 h, 75 °C), and at the same time, VS was degraded. The biogas production from the sonicated and recycled sludge was almost as good as from the pH-adjusted, or heat-treated recycled sludge. After the acid and base treatments of the digested sludge, the recycled sludge yielded often the lowest biogas volume, as the added chemicals increased the FS concentration, which proved to be a more important inhibitory factor than poorly degradable VS. The high FS content significantly reduced the benefits of the treatments. By separating the surface and bottom layers with biogas post-production, the surface layer of VS was concentrated to 51.6–61.8%, while different compositions of the layers affected the biogas production.

## Introduction

The risk of hazardous compounds limits the utilization of sewage treatment plant sludge, biogas production being one of the most common applications (Chen et al. 2008; Chen et al. 2014; Tyagi and Lo 2011). Indeed, anaerobic digestion for thermal and electrical renewable energy purposes has become a well-established technology in the wastewater effluent treatment worldwide. The microbial process converts wastewater sludge volatile solids (VS) into biogas; other possible substrates include manure, energy crops, and municipal solid waste (Appels et al. 2008; Gaida et al. 2017; Weiland 2010). Anaerobic biogas production is a four-stage microbial process consisting of hydrolysis, acidogenesis, acetogenesis/dehydrogenation, and methanation, of which hydrolysis is considered to be the rate-limiting step (Nguyen et al. 2015; Weiland 2010; Feki et al. 2015; Grübel and Suschka 2015). Biogas-producing bacteria mainly belong to phyla Proteobacteria, Firmicutes, and Bacteroidetes, while the most common archaeal bacteria related to methane production belong to orders *Methanomicrobiales*, *Methanosarcinales*, *Thermoplasmatales*, and *Methanobacteriales* (Goswami et al. 2016; Han et al. 2019). The final biogas product consists mainly of methane (60–70% in maximum) and carbon dioxide (Appels et al. 2008).

The VS concentration decreases during the anaerobic digestion due to biogas production, thereby concentrating the inhibitory or even toxic substances of the waste stream and the intermediates of microbial metabolism. The accumulation of inhibitory compounds may eventually prevent biogas production, such as inorganic ions, heavy metals, and ammonia and hydrogen that affect pH (Appels et al. 2008; Chen et al. 2008; Chen et al. 2014). Microorganisms require inorganic ions for growth, though they may affect growth rate and become toxic at high concentrations (Appels et al. 2008; Chen et al. 2008). For heavy metals, toxic concentrations have been estimated (Bååth 1989; Giller et al. 1998). Methanogens are the least tolerant for pH rise due to microbial degradation of proteins and urea to ammonia (Appels et al. 2008; Chen et al. 2008). The optimal pH range for the high solid sludge digestion is between 6.0 and 8.5 (Chen et al. 2007; Lay et al. 1997; Weiland 2010). Nevertheless, the relative importance of these various inhibitory factors in ending biogas production is not well known, although biogas production is a very common process (Zhang and Li 2019).

Biogas-producing microorganisms first consume easily available nutrients and then those that are more difficult to digest (Carrère et al. 2010; Weiland 2010). Therefore, a substantial fraction of the organic material in the final stages of biogas production is poorly microbiologically degradable, consisting of cellulose, hemicellulose, lignin, and other complex organic structures such as hair (Chen et al. 2007; Chen et al. 2008; Tyagi and Lo 2011; Weiland 2010). Enhanced digestion of poorly biodegradable organic compounds at the end of biogas production would enable VS to be recycled back into biogas production and improved recovery of organic material. Techniques that have been reported to improve the hydrolysis of difficult-to-decompose organic fractions and increase bacterial biogas production include acid or alkali treatment, thermal treatment, and sonication (Apul and Sanin 2010; Barber 2016; Carrère et al. 2010; Tyagi and Lo 2011; Zhang et al. 2010; Zhang et al. 2016; Chiu and Lo 2016). However, the effectiveness of these different methods in improving biogas production over, for example, pH control alone is difficult to compare, as different VS hydrolysis techniques have rarely been compared under the same conditions with the same slurry batches. An alternative approach would be the concentrating VS to circumvent the above-presented inhibitory conditions. One such method would be flotation, in which solids are lifted to the surface by attaching to gas bubbles, followed by recovery (Rubio et al. 2002). The possibility of utilizing biogas post-production in flotation (Zeng et al. 2019) to concentrate VS for hydrolysis and recycling back to biogas production has not been studied.

Against this background, the hypothesis of the research was that surface and bottom layer separation with post-produced biogas may concentrate VS and, after digestion, they can be recycled to increase biogas production. Furthermore, it was hypothesized that VS transformation to biogas can be improved by the following post-treatments: pH adjustment, and digesting difficult-to-decompose organic fractions, combined with recycling back into the process. The VS digestion methods used were acid/alkali, thermal, and sonolytic treatments.

## Materials and methods

### Samples and chemical analyses

Low solid sludge (LSS) and dewatered sludge (DS) were collected in Lahti Aqua Ltd. wastewater treatment plant (WWTP, Lahti, Finland), and high solid sludge (HSS) was collected in Labio Ltd. biogas plant (BP, Lahti, Finland), all immediately after biogas production. WWTP produced biogas in low solid process and BP in high solid biogas production process. The sewage sludge was used as a raw material in both processes, and BP mixed the source-separated biowaste with the sewage sludge in a ratio of about 3:1. For experiment 1 (E1), the LSS was aerated after biogas production, but not for experiment 2 (E2). Aeration reduces microbial biogas-producing activity. Samples for E1 were collected on November 29, 2017, and those for E2 on February 7, 2018. On the sampling day, the sludges were weighed for total (TS), fixed (FS), and volatile (VS) solid analyses; for pH measurements; element analyses, surface, and bottom layer separations; and for measuring time-dependent changes in sludge pH (experiment 3, E3). Then, the sludges were frozen at − 20 °C for biogas production experiments.

TS (heating for 20 h at 105 °C), FS, and VS (heat treatment for 4 h at 550 °C) of the sludges were determined in triplicate as presented earlier (Kerminen et al. 2018). To measure pH, 1.0 g of sludge was mixed with 4 mL of distilled water, and the mixture was shaken for 1 h at 150 rpm (Laboshake; Gerhardt, Königswinter, Germany). Then, the liquid was separated by centrifugation at 2027 rpm (Heraeus 1S-R with 75002002 rotor, DJB Labcare, Buckinghamshire, UK; 3000×*g*, 10 min), and pH was measured using InoLab series pH 720 m (Weilheim, Germany) (Kurola et al. 2011). Alternatively, pH was measured using the pH paper (Fisher Scientific, Hampton, NH, USA). Carbon and nitrogen were determined using LECO CNS-2000 elemental analyzer (LECO Corporation, St. Joseph, MI, USA) as presented previously (Talja et al. 2008). To measure the elements Al, Co, Cr, Cu, Mn, Ni, Fe, Zn, P, V, and Pb, the sludge was treated with nitric acid in a MARS 6 microwave digestion system according to the manufactures instructions (CEM Corporation, Matthews, NC, USA). Then, the samples were diluted with water to a nitric acid concentration of 2% (vol/vol), and the elements were measured using the Sciex Elan 6000 ICP-MS equipment (Perkin Elmer Inc., Waltham, MA, USA). The method is based on standards (SFS-ISO 17294-1 2004; SFS-EN ISO 17294-2 2016).

### Surface and bottom layer separation

For E1, to separate surface and bottom layers by biogas post-production, 2.0 L of LSS (wet weight (wt) 2010 g; TS, 51 g) was transferred to a 6-L plastic container. A 1.0-L volume of DS (wet wt, 600 g; TS, 159 g) and HSS (wet wt, 885 g; TS, 175 g) was mixed with 1.5 L of distilled water in 6-L plastic containers. The sludges in triplicate were incubated at the room temperature of 21 ± 2 °C for 40 days. The surface layers were collected, water was separated by centrifugation at 2027 rpm (3000×*g*, 10 min), and the sludges were frozen at − 20 °C for the biogas production experiment.

For E2, to separate surface and bottom layers, 2.0 L of LSS (wet wt, 1930 g; TS, 70 g) was transferred to a 3-L plastic container. DS (wet wt, 600 g; TS, 161 g) and HSS (wet wt, 885 g; TS, 208 g) of 1.0 L were mixed with 1.5 L of distilled water and incubated in 3-L plastic containers, all at the room temperature of 21 ± 2 °C in triplicate. The surface and bottom layers were collected after 28 (HSS, surface layer 7.4 g dry wt, bottom layer 281 g dry wt) and 33 days (LSS, surface layer 10.3 g dry wt; bottom layer 89.6 g dry wt; DS surface layer, 32.9 g dry wt; bottom layer, 320 g dry wt); water was removed by centrifugation at 2027 rpm (3000×*g*, 10 min), and the sludges were frozen at − 20 °C for the biogas production experiment. To follow the surface and bottom layer separation, another set of similar incubations in triplicate was done from DS and HSS, and the samples were collected from bottom and surface layers after 5, 12, 19, 26, and 28 days.

### Biogas production

All biogas measurements were done in triplicate, and biogas yield was calculated as mL of biogas/g of VS on a dry weight basis. In E1, to measure biogas production, the methane-producing microbial community of the WWTP was transferred to 20-mL syringes in 10 mL of LSS, with and without pH 7.0 adjustments. Then acid- or alkali-treated and acid- or alkali-neutralized sludges were recycled to the LSS with pH 7.0. The LSS properties were as follows: pH, 9.0; TS, 2.5 ± 0.1%; VS, 45.6 ± 0.1% of TS; FS, 54.4 ± 0.1% of TS. The quantities of TS, VS, and FS in the sludges of E1 and the VS substrate to inoculum ratios (*S/I* ratios) are presented in Table 1. The average of VS *S/I* ratios was 0.76 ± 0.31 in E1, and the ratio ranged between 0.41 and 1.24; i.e., changes in the ratios may have had minor effects on biogas production, which has been the best close to *S/I* ratio of 1:1 (Córdoba et al. 2018). Biogas production was monitored from the increase in gas volume in an airtight syringe with a flexible piston for 24 days at 37 °C (model C25KC incubator shaker, New Brunswick Scientific Co., Edison, NJ. USA). In E2, to measure biogas production, the WWTP methane-producing microbial community was transferred to a 20–50-mL syringe in 5 mL of LSS, with and without pH 7.0 adjustment. The treated sludges (acid/alkali treatment, thermal treatment, sonication) were recycled to the LSS with pH 7.0. The LSS properties were as follows: pH, 7.9; TS, 3.7 ± 0.1%; VS, 54.6 ± 9.1% of TS; FS, 45.4 ± 9.1% of TS. The quantities of TS, VS, and FS in the sludges of E2, and the VS *S/I* ratios are presented in Table 1. The average VS *S/I* ratios were 1.52 ± 0.89 in E2, and the ratio ranged between 0.54 and 2.90. Biogas production at 37 °C and under 30 rpm shaking was followed for 14 days.

**Table 1:** Total solid (TS), volatile solid (VS), and fixed solid (FS) concentrations; wet weights; and/or substrate to inoculum VS ratios (*S/I* ratio) of sludges in treatments, and in biogas production experiments. Values are presented as average ± S.D (*n* = 3)

**Sludges in treatments**	**Wet weight**	**TS (g dry weight)**	**VS (g dry weight)**	**FS (g dry weight)**
Experiment 1 (E1)				
Low solid sludge (LSS)	10 mL	0.251 ± 0.009	0.115 ± 0.004	0.136 ± 0.005
Surface layer of LSS (SLSS)	1.0 g	0.092 ± 0.004	0.047 ± 0.001	0.045 ± 0.003
Dewatered sludge (DS)	1.0 g	0.263 ± 0.001	0.142 ± 0.001	0.121 ± 0.001
Surface layer of DS (SDS)	1.0 g	0.143 ± 0.007	0.076 ± 0.004	0.067 ± 0.003
High solid sludge (HSS)	1.0 g	0.197 ± 0.003	0.091 ± 0.001	0.106 ± 0.003
Surface layer of HSS (SHSS)	1.0 g	0.151 ± 0.061	0.071 ± 0.013	0.080 ± 0.072
Experiment 2 (E2)				
Low solid sludge (LSS)	5.183	0.183 ± 0.002	0.099 ± 0.016	0.083 ± 0.017
Surface layer of LSS (SLSS)	1.305	0.100	0.053 ± 0.001	0.047 ± 0.001
Bottom layer of LSS (BLSS)	1.044	0.100	0.053 ± 0.001	0.047 ± 0.001
Dewatered sludge (DS)	1.869	0.500	0.283 ± 0.002	0.217 ± 0.002
Surface layer of DS (SDS)	1.616	0.200	0.111 ± 0.002	0.089 ± 0.002
Bottom layer of DS (BDS)	1.570	0.200	0.111 ± 0.001	0.089 ± 0.001
High solid sludge (HSS)	2.125	0.500	0.208 ± 0.008	0.292 ± 0.008
Surface layer of HSS (SHSS)	1.463	0.200	0.103 ± 0.013	0.097 ± 0.013
Bottom layer of HSS (BHSS)	1.412	0.200	0.163 ± 0.048	0.337 ± 0.048
**Sludges in biogas production experiments**	**S/I ratio**	**TS (g dry weight)**	**VS (g dry weight)**	**FS (g dry weight)**
Experiment 1 (E1)				
Low solid sludge (LSS)		0.251 ± 0.009	0.115 ± 0.004	0.136 ± 0.005
Surface layer of LSS (SLSS)	0.41 ± 0.01	0.343 ± 0.011	0.162 ± 0.005	0.181 ± 0.006
Dewatered sludge (DS)	1.24 ± 0.04	0.514 ± 0.009	0.257 ± 0.004	0.257 ± 0.005
Surface layer of DS (SDS)	0.66 ± 0.03	0.394 ± 0.014	0.190 ± 0.007	0.204 ± 0.007
High solid sludge (HSS)	0.79 ± 0.03	0.448 ± 0.010	0.206 ± 0.003	0.242 ± 0.007
Surface layer of HSS (SHSS)	0.62 ± 0.13	0.402 ± 0.070	0.186 ± 0.011	0.216 ± 0.076
Experiment 2 (E2)				
Low solid sludge (LSS)		0.183 ± 0.002	0.100 ± 0.016	0.083 ± 0.017
Surface layer of LSS (SLSS)	0.54 ± 0.08	0.282 ± 0.002	0.152 ± 0.017	0.130 ± 0.017
Bottom layer of LSS (BLSS)	0.54 ± 0.09	0.283 ± 0.002	0.152 ± 0.016	0.131 ± 0.017
Dewatered sludge (DS)	2.90 ± 0.46	0.683 ± 0.002	0.383 ± 0.018	0.300 ± 0.019
Surface layer of DS (SDS)	1.14 ± 0.20	0.383 ± 0.002	0.211 ± 0.015	0.172 ± 0.015
Bottom layer of DS (BDS)	1.14 ± 0.19	0.383 ± 0.002	0.211 ± 0.016	0.172 ± 0.017
High solid sludge (HSS)	2.13 ± 0.34	0.683 ± 0.002	0.308 ± 0.019	0.375 ± 0.020
Surface layer of HSS (SHSS)	1.04 ± 0.04	0.383 ± 0.002	0.203 ± 0.029	0.180 ± 0.030
Bottom layer of HSS (BHSS)	1.72 ± 0.74	0.683 ± 0.002	0.263 ± 0.032	0.420 ± 0.032

### Digested sludge post-treatments to improve biogas production

All experiments were done in triplicate. TS, VS, FS, and wet weights of sludges in the treatments were as presented in Table 1. In E1, the LSS volume was 10 mL (digested sludge collected after aeration) in biogas production experiments, and the sludge treatments in the first experiment were pH 7.0 adjustment and acid and alkali hydrolysis. In E2, the LSS volume was 5 mL (digested sludge collected prior to aeration) in biogas production experiments, and the treatments were as follows: pH 7.0 adjustment; acid and alkali hydrolysis; thermal treatment; and sonication.

#### pH adjustment

In E1, the initial pH values of the LSS, DS, and HSS were 9.0, 8.0, and 10.0, respectively. Biogas production in the sludges (LSS bacterial inoculum 10 mL) was measured with and without pH 7.0 adjustment. In E2, the initial pH values of the LSS, SLSS, BLSS, DS, SDS, BDS, HSS, and SHSS, and BHSS was 7.9, 8.7, 8.7, 8.8, 7.4, 7.4, 8.5, 8.3, and 8.3, respectively. Biogas production in the sludges (LSS bacterial inoculum 5 mL) was measured with and without pH 7.0 adjustment. In E1 and E2, the pH 7.0 was adjusted with 37% hydrochloric acid (HCl), and pH was measured using pH paper. In E3, to monitor whether the sludge pH could be lowered to 7.0 during storage at the room temperature of 21 ± 2 °C without acid addition, DS (dry wt 26.8 g) and HSS (dry wt 23.5 g) with the wet wt of 100 g were transferred in a 0.5-L decanter class covered with an aluminum foil. The samples of 1.0 g were taken after 0, 1, 2, 5, 6, 12, 32, and 35 days for the pH measurement. The pH was measured using InoLab series pH 720 m as presented above.

#### Acid and alkaline hydrolysis

To study the effects of acid hydrolysis of digested sludge on biogas production, the pH value of sludge was adjusted to pH 1.0, 2.0 or 3.0 using 37% HCl, and the solutions were incubated at the room temperature of 21 ± 2 °C for 24 h (Devlin et al. 2011). To study the effects of alkaline hydrolysis of digested sludge on biogas production, the pH values of sludges were adjusted to pH 10.0 or 12.0 using 10 M potassium hydroxide (KOH), and the solution was incubated at the room temperature of 21 ± 2 °C for 48 h (Rafique et al. 2010). After the treatments, the samples were neutralized; i.e., pH 7.0 was adjusted using HCl or KOH.

In E1, the acid-treated (pH 1.0, 2.0, and 3.0) or alkaline-treated (pH 10.0, and 12.0) sludges were recycled to biogas production; i.e., the sludges were amended in 10 mL of LSS to measure biogas production. The acid- or alkaline-treated sludge samples were SLSS (pH 1.0 and 12.0), DS (pH 1.0 and 12.0), SDS (pH 1.0, 2.0, 3.0, 10.0 and 12.0), HSS (pH 1.0 and 12.0), and SHSS (pH 1.0 and 12.0). In E2, the acid-treated (pH 3.0) or alkaline-treated (pH 10.0) sludges were recycled to biogas production; i.e., the sludges were amended in 5 mL of LSS to measure biogas production. The acid- or alkaline-treated sludge samples were SLSS (pH 10.0), and BLSS (pH 10.0), DS (pH 3.0 or 10.0), SDS (pH 10.0), BDS (pH 10.0), HSS (pH 3.0 and 10.0), SHSS (pH 10.0), and BHSS (pH 10.0).

#### Thermal treatment (heating) and sonication

In E2, to disrupt the sludge microbial cells and VS, and to change sludge rheology, the sludges were heated in a water path (Grant SUB 14, Cambridge, UK) at 75 °C until the pH fell to 7.0 due to ammonia evaporation (Barber 2016; Bonmatí and Flotats 2003; Climent et al. 2007; Tyagi and Lo 2011). The incubation time varied between 1 and 3 h. The heat-treated sludges were amended in 5 mL of LSS to measure biogas production. The heat-treated sludges were SLSS (3 h), BLSS (3 h), DS (3 h), SDS (2 h), BDS (2 h), HSS (2 h), SHSS (1 h), and BHSS (1 h).

The high pressure created by sonication causes microbubble formation, which collides and releases energy for radical formation and biological material disruption (Apul and Sanin 2010). For sonolytic treatment, the sludges were sonicated for 60 min at 43 kHz and 320 W (Branson 8510 Ultrasonic, Danbury, USA) until the pH fell to 7.0. The sonicated sludges were amended in 5 mL of LSS and biogas production was measured. The sonicated sludges were DS and HSS.

### Calculations

The results were calculated as an average ± standard deviation (S.D.) (*n* = 3). Statistical analyses were performed using IBM SPSS Statistics 24 (New York, USA). The repeated measures ANOVA (RMA) followed by pairwise comparisons (PC) was used to determine whether the VS concentration differed between the surface and bottom layers of DS and HSS and whether the sludge pH changed during storage. The principal component analysis (PCA) was used to elucidate the distribution of inorganic ions into the surface and bottom layers of DS and HSS. Two-factor (sludge, treatment) Kruskal-Wallis (KW) test followed by Mann-Whitney’s test (MW) was used to determine whether sludge compositions and treatments had an effect on biogas yield.

## Results and discussion

The LSS total solid content of 3.1 ± 0.6% of wet wt was low at the end of WWTP biogas production process. It was increased by centrifugation to 26.6 ± 0.31% of the DS, while the total solid content of the BP HSS was 21.6 ± 2.2% after biogas production. The percentages of VS in the LSS (E1, 45.6 ± 0.1% of dry wt; E2, 54.6 ± 9.1%), DS (E1, 54.1 ± 0.1%; E2, 56.6 ± 0.4%), and HSS (E1, 46.3 ± 0.8%; E2, 41.7 ± 1.7%) were still quite high at the end of biogas production. The pH values of the LSS (E1 9.0/E2 7.9), DS (E1 8.0/E2 8.8), and HSS (E1 10.0/E2 8.5) were close to the upper limit of biogas-producing bacteria (about pH 8.5) or higher, most likely due to ammonia release from proteins and urea (Appels et al. 2008; Chen et al. 2007; Lay et al. 1997; Weiland 2010) (Table 2).

**Table 2:** Volatile solids (VS), total C, total N, pH values, and element concentrations in high solid sludge (HSS), surface layer of high solid sludge (SHSS), bottom layer of high solid sludge (BHSS), dewatered sludge (DS), surface layer of dewatered sludge (SDS), and bottom layer of dewatered sludge (BDS). Values (average ± S.D.) are presented on a dry weight basis and per VS; < LOD, below the limit of detection

	HSS (per dry wt)	HSS (per VS)	SHSS (per dry wt)	SHSS (per VS)	BHSS (per dry wt)	BHSS (per VS)	DS (per dry wt)	DS (per VS)	SDS (per dry wt)	SDS (per VS)	BDS (per dry wt)	BDS (per VS)
VS (%)	41.7 ± 1.7		51.6 ± 6.6		32.7 ± 9.6		56.6 ± 0.4		55.5 ± 0.9		55.5 ± 0.1	
Total C (g/kg)	209.7 ± 12.3	503.1 ± 28.6	236.5 ± 11.2	465.3 ± 79.7	157.3 ± 9.0	508.5 ± 138.4	305.3 ± 33.5	539.5 ± 62.5	ND^a^	ND^a^	ND^a^	ND^a^
Total N (g/kg)	27.3 ± 1.0	65.7 ± 4.4	31.7 ± 2.2	62.2 ± 10.3	15.9 ± 2.0	50.7 ± 10.0	46.1 ± 5.7	81.5 ± 10.6	ND^a^	ND^a^	ND^a^	ND^a^
pH	8.5	8.5	8.3	8.3	8.3	8.3	8.8	8.8	7.4	7.4	7.4	7.4
Al (g/kg)	22.3 ± 5.1	53.5 ± 12.3	39.5 ± 6.2	76.6 ± 12.0	35.3 ± 2.0	108.1 ± 6.1	3.1 ± 0.2	5.5 ± 0.3	4.0 ± 0.1	7.2 ± 0.1	4.8 ± 0.3	8.7 ± 0.5
Fe (g/kg)	20.4 ± 5.4	48.9 ± 13.0	39.1 ± 3.6	75.9 ± 7.0	26.8 ± 1.6	82.2 ± 4.9	151.4 ± 7.9	267.4 ± 14.0	185.8 ± 0.1	334.8 ± 0.1	227.6 ± 0.1	410.2 ± 0.1
P (g/kg)	9.6 ± 2.7	22.9 ± 6.5	15.0 ± 2.6	29.1 ± 5.0	6.8 ± 0.4	20.8 ± 1.2	35.7 ± 2.4	63.1 ± 4.2	59.4 ± 0.6	107.0 ± 1.0	49.8 ± 0.6	89.8 ± 1.0
Co (mg/kg)	< LOD-3.4	< LOD-8.2	5.5 ± 0.6	10.6 ± 1.2	< LOD-3.1	< LOD-9.5	9.0 ± 0.5	15.8 ± 0.9	10.5 ± 0.1	18.9 ± 0.1	13.3 ± 0.1	24.0 ± 0.1
Cr (mg/kg)	17.2 ± 2.1	41.3 ± 5.0	30.0 ± 2.1	58.1 ± 4.0	21.3 ± 1.4	65.3 ± 4.3	15.3 ± 1.1	27.1 ± 1.9	19.8 ± 0.6	35.7 ± 1.0	24.3 ± 3.3	43.9 ± 6.0
Cu (mg/kg)	43.6 ± 4.5	104.5 ± 10.8	91.4 ± 5.2	177.3 ± 10.0	33.9 ± 4.0	103.8 ± 12.2	201.9 ± 10.6	356.6 ± 18.7	246.4 ± 5.7	444.0 ± 10.3	294.3 ± 5.6	530.5 ± 10.0
Mn (mg/kg)	136.0 ± 24.0	326.2 ± 57.7	234.1 ± 20.7	453.8 ± 40.1	137.0 ± 2.0	419.6 ± 6.1	579.4 ± 26.4	1023.5 ± 46.7	690.6 ± 5.7	1244.6 ± 10.3	863.4 ± 0.1	1556.0 ± 0.1
Ni (mg/kg)	13.6 ± 0.6	32.6 ± 1.4	17.6 ± 2.1	34.0 ± 4.0	13.3 ± 2.0	40.7 ± 6.1	27.7 ± 3.2	48.9 ± 5.6	31.5 ± 2.3	56.8 ± 4.1	40.4 ± 0.6	72.9 ± 10.0
Zn (mg/kg)	< LOD-119.0	< LOD-285.4	252.4 ± 5.2	489.3 ± 10.0	< LOD-110.2	< LOD-337.4	616.8 ± 26.4	1089.5 ± 46.7	743.1 ± 0.1	1339.3 ± 0.1	902.7 ± 55.5	1626.8 ± 100.0
V (mg/kg)	23.8 ± 4.8	57.1 ± 11.5	41.3 ± 6.7	80.1 ± 13.0	36.7 ± 4.0	112.5 ± 12.2	13.1 ± 0.5	23.1 ± 0.9	16.2 ± 0.1	29.1 ± 0.1	19.6 ± 0.1	35.4 ± 0.1
Pb (mg/kg)	6.4 ± 1.2	15.3 ± 2.9	13.5 ± 0.5	26.2 ± 1.0	8.9 ± 1.0	27.3 ± 3.1	6.0 ± 0.1	10.6 ± 0.1	7.3 ± 0.2	13.2 ± 0.3	9.4 ± 0.1	17.0 ± 0.1

^a^*ND*, not determined

As the factors like high element/FS concentration, high pH, and difficult-to-digest VS composition are suggested to limit biogas production (Appels et al. 2008; Chen et al. 2008), the importance of these factors in reducing biogas yields to below profitable levels at VS concentrations as high as 41.7–56.6% was evaluated. Furthermore, the possibility to concentrate the VS of the LSS, DS, and HSS by lifting onto the surface with biogas post-production and to digest them into more suitable substrates for biogas-producing bacteria was studied.

### Surface and bottom layer separation

**Fig. 1:**
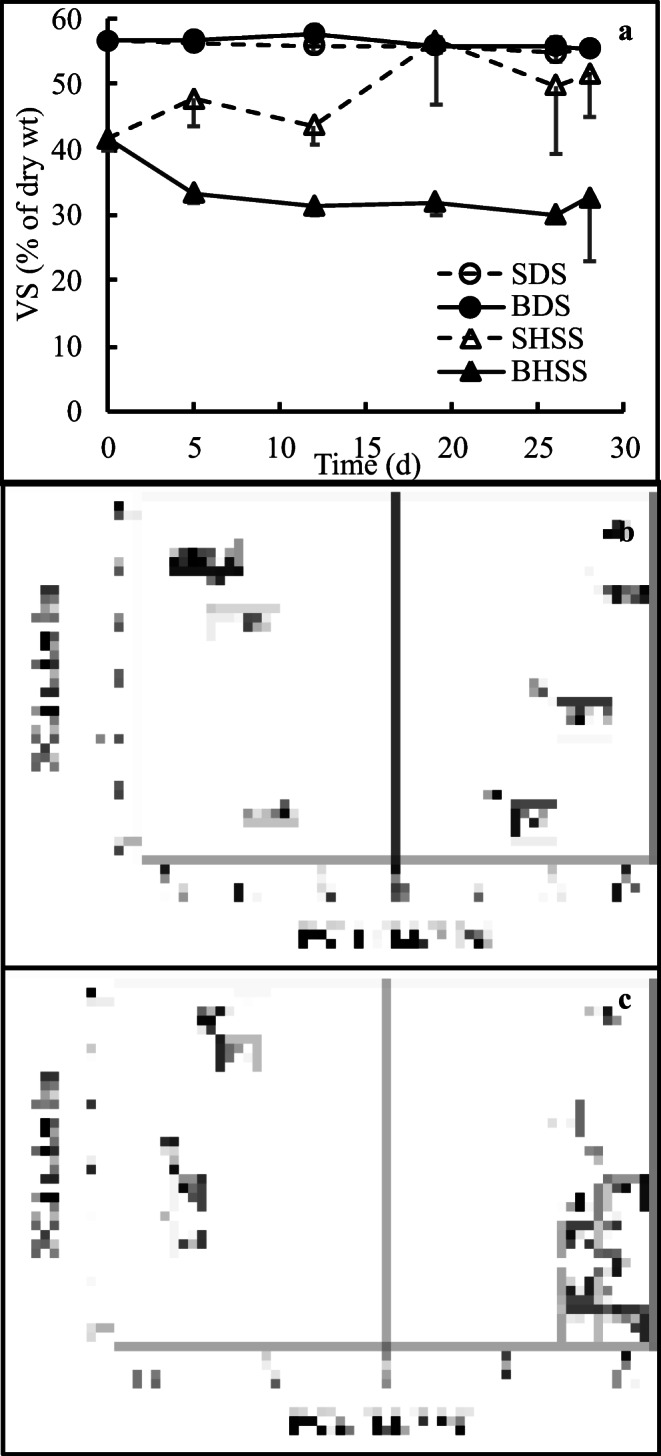
**a** Volatile solid (VS) content in the dewatered sludge (DS) surface (SDS) and bottom (BDS) layers, and in the high solid sludge (HSS) surface (SHSS) and bottom (BHSS) layers, separated by biogas post-production (*n* = 3; the average of standard deviations, 2.2%). **b** Principal component analysis score plot showing the separation of DS, SDS, BDS, HSS, SHSS, and BHSS along the PC1 and PC2 axes based on the elemental compositions. **c** Loading values for elements along the PC1 and PC2 axes

The feasibility of concentrating VS by raising to the surface using biogas post-production was studied, as gases are known to carry suspended matter on the water surface (Rubio et al. 2002). In E1, the initial VS concentration of 46.3 ± 0.8% for HSS increased to 61.8 ± 3.6% in the surface layer and decreased to 42.9 ± 7.3% in the bottom layer, the difference being 18.9% in 40 days. Then, in E2, the changes in the VS concentration were followed during the incubation (Fig. 1a). The VS concentration in the surface layer of HSS increased significantly from 41.7 ± 1.7% to 51.6 ± 6.6% in 28 days, while the bottom layer VS concentration decreased to 32.7 ± 9.6% (RMA-PC, *p* = 0.001). The greatest difference in VS concentration between the surface and bottom layers was achieved in 19 days, and it was again as much as 18.9% on a dry weight basis. Based on total C, 60.1% (236.5 ± 11.2 g/kg) of total C was accumulated on the surface of HSS and 39.9% (157.3 ± 9.0) on the bottom, the difference of 20.1% being close to that calculated on the basis of VS (Table 2). In contrast to HSS, the VS concentration for DS did not differ between the surface and bottom layers (E1: initial 54.1 ± 0.1%, end 54.0 ± 1.4%; E2: initial 56.6 ± 0.4%, end 55.5 ± 0.9%, RMA-PC, *p* = 0.161). Similarly, the VS concentration for LSS did not differ between the surface and bottom layers (E2: initial 52.9 ± 0.6%, end 52.5 ± 0.5%). Thus, the highest percentage of VS that could be accumulated on the surface layer varied between 51.6 and 61.8% of dry weight (Fig. 1a; Table 2).

The quantity of sludge brought to the surface by biogas post-production was low, only about 2.6% of HSS, and 10.3% of DS based on the dry weight. However, all large solids were removed from the surface before weighting, which leads to an underestimation of the separation efficiency especially in HSS, which contained source-separated biowaste with plastic bag residues. Elemental concentrations in the surface and bottom layers of HSS and DS were usually equal to or higher than in the sludges prior to separation the layers, which indicates that part of the VS may have been lost during the layer separation process as volatile biogas, or as dissolved in the liquid phase due to water addition (Table 2).

The major differences in the elemental compositions per VS were due to differences between HSS and DS, covering 82.7% of variance in the PCA (Fig. 1b). The concentrations of Fe, P, Co, Cu, Mn, Ni, and Zn in DS were higher than in HSS, while those of Al and V were highest in HSS (Fig. 1c and Table 2). Cr and Pb concentrations did not differ much between HSS and DS. Differences in elements between sludges, surface layers, and bottom layers accounted for 15.0% of variance along the PCA PC2 axis (Fig. 1b, c). Elemental concentrations per VS had a tendency to increase in the following order: Sludge < surface < bottom (Table 2). Their concentrations were among the highest in the DS bottom layer, where the VS content was almost the same at the surface and bottom. However, the P, Cu, Mn, and Zn concentrations were highest in the surface layer of HSS (VS, 51.6%), which had much more VS to adsorb elements than the bottom layer (VS, 32.7%).

Although elemental concentrations were affected by surface and bottom layer separation, still quite high concentrations were found in both layers. Especially Cu and Zn concentrations were high enough to inhibit microbial activity in all sludges (Bååth 1989), and increasing elemental concentrations generally increase osmotic pressure. The surface and bottom layer separation was not very efficient in concentrating the elements, but it may be possible to use to increase the surface layer VS concentration relative to the bottom. Factors that are known to affect the surface and bottom layer separation include the efficiency of methane post-production, water quantity, substances dissolved in water, sludge composition, and pH (Rubio et al. 2002). For example, Cr, Cu, Ni, and Zn are released from the sludge into the liquid at low pH values below 6.3 (Adams and Sanders 1984; Wong et al. 2002). The optimization of these conditions could improve the separation efficiency. In addition, surface and bottom layer separation immediately at the end of biogas production process could be more efficient, and the biogas produced during surface separation could be recovered. The activity of the biogas-producing microbial community would not be affected by aeration as a post-treatment, transportation, and storage under aerobic conditions for up to 24 h prior to the start of experiments, and freezing, as was in this study.

### Digested sludge post-treatments to improve biogas production

To improve biogas production from recycled digestate, the pH of the digestate was adjusted to 7.0, which is optimal for methane-producing bacteria (Appels et al. 2008; Chen et al. 2008). In addition, sludge VS were hydrolyzed by acid, alkali, heat, or sonolytic treatment, followed by recycling to biogas production. Based on statistical analysis using KW, the treatments (*p* < 0.001) and recycled materials (*p* < 0.050) affected especially biogas production, as shown below.

#### Digested sludge pH adjustment

**Fig. 2:**
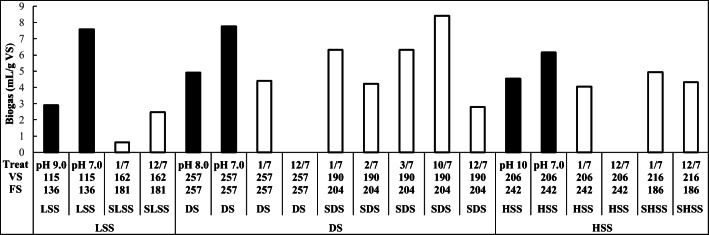
Treatments (Treat), and volatile (VS) and fixed (FS) solid contents (g/kg dry wt) in experiment 1 (E1). Black bars: Biogas production in LSS (pH 9.0, pH 7.0), and in LSS amended with DS (pH 8.0, pH 7.0) or HSS (pH 10.0, pH 7.0); the first value without pH adjustment, and the second with pH 7.0 adjustment. White bars: Biogas production in LSS amended with acid-treated (treatment pH/biogas production pH: pH 1/7, 2/7, and 3/7) or alkali (pH 10/7, 12/7) SLSS, DS, SDS HSS, or SHSS, followed by neutralization For all treatments, the average of standard deviations was 0.61 mL/g VS (*n* = 3)

**Fig. 3:**
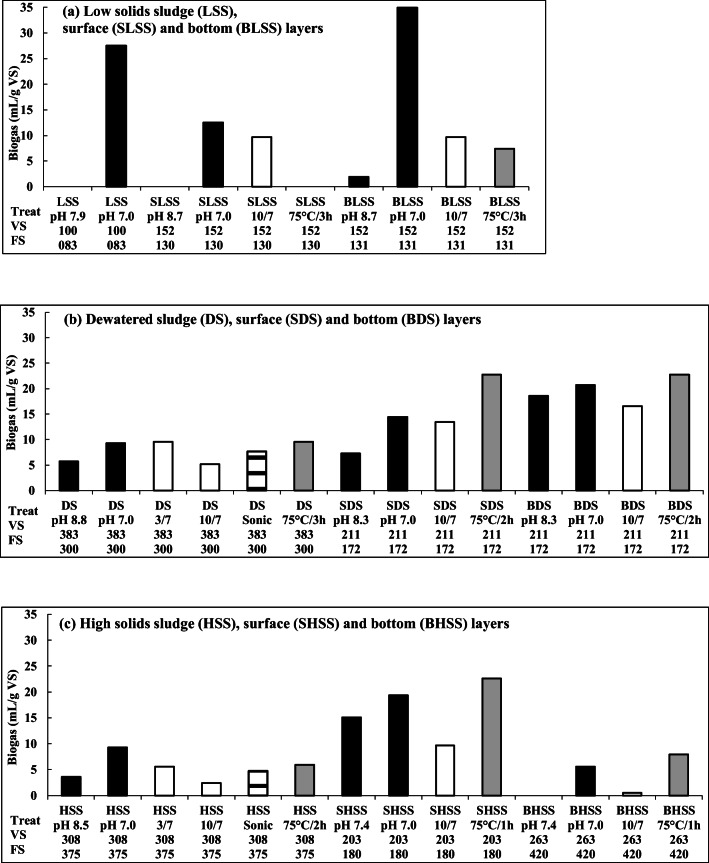
E2, treatments (Treat, *n* = 3), and volatile (VS) and fixed (FS) solid contents (g/kg dry wt). **a** Black bars: Biogas production in LSS (pH 7.9, pH 7.0), and in LSS amended with SLSS (pH 8.7; pH 7.0) or BLSS (pH 8.7, pH 7.0) without and with pH 7.0 adjustment. White bars: Biogas production in LSS amended with alkali-treated (treatment pH/biogas production pH: pH 10/7) SLSS or BLSS, followed by neutralization. Gray bars: Biogas production in LSS amended with temperature-treated (75 °C/3 h) SLSS or BLSS. **b** Black bars: Biogas production in LSS amended with DS (pH 8.8, pH 7.0), SDS (pH 8.3, pH 7.0), or BDS (pH 8.3, pH 7.0) without and with pH adjustment. White bars: Biogas production in LSS amended with acid-treated (3/7) or alkali-treated (10/7) DS, SDS, or BDS, followed by neutralization. Gray bars: Biogas production in LSS amended with temperature-treated DS (75 °C/3 h), SDS, or BDS (75 °C/2 h). Horizontal line bars: Biogas production in LSS amended with sonicated DS. **c** Black bars: Biogas production in LSS amended with HSS (pH 8.5, pH 7.0), SHSS (pH 7.4; pH 7.0), or BHSS (pH 7.4, pH 7.0) without and with pH adjustment. White bars: Biogas production in LSS amended with acid-treated (3/7) or alkali-treated (10/7) HSS, SHSS, or BHSS, followed by neutralization. Gray bars: Biogas production in LSS amended with temperature-treated HSS (75 °C/2 h), SHSS, or BHSS (75 °C/1 h). Horizontal line bars: Biogas production in LSS amended with the sonicated HSS. For all treatments, the average of standard deviations was 3.3 mL/g VS (*n* = 3)

In E1 and E2, the pH of the LSS, DS, and HSS, and their surface and bottom layers was adjusted to pH 7.0 from the pH values of 7.4–10.0, and then the sludges were recycled to biogas production. Biogas production was compared with that in the same recycled sludge without adjusted pH (Figs. 2 and 3, black bars). Generally, the recycling of non-pH-adjusted sludges to biogas production increased biogas yield compared with LSS without pH adjustment, except not the SLSS (Fig. 3a) and BHSS (Fig. 3c) recycling. Similarly, according to Yadvika et al. (2004), the sole recirculation of sludge back to biogas production has increased biogas yield, but only marginally. Biogas yield was further increased, when the pH of the sludges was adjusted to 7.0 (MW, *p* < 0.001; Figs. 2 and 3, black bars). Indeed, adjusting the pH of the microwave H_2_O_2_ pre-treated sludge also improved biogas production (Eswari et al. 2016). Thus, ammonia release from proteins and urea appeared to raise the pH of sludges above the optimum of biogas production (Appels et al. 2008; Chen et al. 2008). Adjusting the pH of the recycled digested sludge was one of the most effective methods for achieving a high biogas yield.

**Fig. 4:**
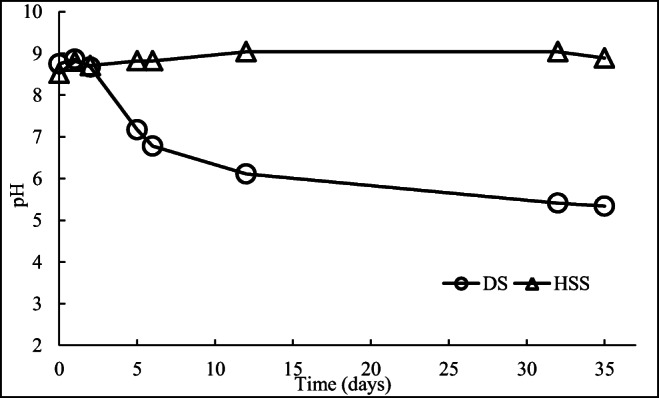
The changes in the pH of dewatered (DS) and high solid (HSS) sludges during the incubation at the temperature of 21 ± 2 °C for 35 days (the average of standard deviations, 0.05; *n* = 3)

In E3, the pH of DS and HSS was monitored for 35 days to determine if it could be lowered to pH 7.0 during storage without the addition of acid or base. The water evaporated during incubation, as dry weight increased from 26.8 to 48.2% in DS (RMA-PC, *p* = 0.002) and from 23.5 ± 0.8 to 77.1 ± 17.7% in HSS (RMA-PC, *p* = 0.002). At the same time, the pH of the coarse and aerobic DS decreased from the initial pH 8.8 to pH 6.8–7.2 in 5–6 days, and further to 5.3 in 35 days (RMA-PC; *p* < 0.001) (Fig. 4). For comparison, in the surface and bottom layer separation, the pH of DS with added water only decreased from 8.8 to 7.4 during 33 days of incubation under saturated conditions (Fig. 1, Table 2). During DS storage in E3, the decrease in pH was associated with only a 2.9% decrease in total N, from 46.1 ± 5.7 to 44.9 ± 1.2 g/kg (RMA-PC, *p* = 0.003) (Fig. 4). Total C decreased more in E3, 29.8% from 305.3 ± 33.5 to 214.3 ± 12.0 g/kg (RMA-PC, *p* < 0.001), while the VS percentage remained almost the same (57.1 ± 0.6% of dry wt) for 35 days. Ammonia released from proteins and urea appeared to be microbiologically bound to biomass in coarse and aerobic DS, as only little nitrogen was lost even though the pH decreased. Simultaneously some carbon was lost in cellular respiration.

In E3, the pH changes in HSS differed from those in DS. The pH of HSS increased from an initial pH of 8.5 to 8.9 during a 35-day incubation (RMA-PC, *p* < 0.001), while total N decreased by 61.9% from 27.3 ± 1.0 g/kg to 10.4 ± 0.7 g/kg (RMA-PC, *p* = 0.003). Total C decreased as much as 64.5%, from 209.7 ± 12.3 g/kg to 74.4 ± 5.4 g/kg (RMA-PC, *p* ≤ 0.001), though these changes were not clearly seen in the VS content (initial 41.7 ± 1.7%; final 48.6 ± 15.7% of dry wt). Thus, under saturated conditions, biogas production at HSS seemed to continue and ammonia was released further, which was reflected in the increase in pH and the removal of some of the ammonia by evaporation. Altogether, under aerobic conditions at about 21 °C, ammonia in the digested sludge seemed to be incorporated to biomass and pH decreased to 7.0 in a few days, but under saturated conditions, the ammonia will remain in solution or evaporate slightly so that the pH will not decrease. To remove ammonia from the system, for instance, stripping of ammonia is required (Bonmatí and Flotats 2003).

#### Digested sludge acid, alkali, thermal, and sonolytic treatments

In E1, the sludges were treated with acid (pH 1.0, 2.0, or 3.0) or alkaline (pH 10.0, or 12.0) and then recycled to biogas production at pH 7.0 (Fig. 2, white bars). According to the results, the biogas yields after the pH 1.0 and 12.0 treatments of the recycled sludges were lower than after the pH 7.0 adjustments (MW, *p* < 0.001). The biogas yields were the best after the pH 1.0, 3.0, and 10.0 treatments of the recycled sludges (*p* ≤ 0.050), the pH 10.0 treatment being the best (MW, *p* = 0.050), as also reported in Chen et al. (2007), Zhang et al. (2010), and Feki et al. (2015). The ability of pH 3.0 and 10.0–treated recycled sludges to improve biogas production was further investigated in E2 (Fig. 3, white bars). The results showed that the recycling of pH 3.0 and 10.0–treated sludges improved the biogas yield less than the pH 7.0 adjustment alone (MW, *p* ≤ 0.021).

Digested sludge VS heat and sonolytic hydrolyses were also studied in E2. The recycled slurry was heated at 75 °C or sonicated until the pH fell to 7.0 due to ammonia evaporation (Fig. 3, gray bars). According to the results, the heat treatment of the recycled sludges improved the biogas yield as much as the pH 7.0 adjustment (MW, *p* = 0.304), in agreement with other studies stating that thermal treatment is one of the most effective pre-treatments (Bougrier et al. 2006; Hao et al. 2016; Kim et al. 2003; Pérez-Elvira et al. 2006). Thus, the ammonia recovery by heat stripping would at the same time digest the VS more susceptible to microbial use and recyclable back to biogas production (Bonmatí and Flotats 2003). The biogas yields after 1–2-h heating of the recycled sludges were among the highest (Fig. 3b, c, gray bars), while 3-h heating appeared to have adverse effects on biogas production (Fig. 3a, gray bars). The biogas yields from sonicated and recycled DS and HSS did not differ from the yields after heat treatment of the sludges (MW, *p* = 0.108), but sonication was less efficient treatment than pH 7.0 adjustment (MW, *p* = 0.003) (Fig. 3b, c, horizontal line bars).

#### Digested sludge materials

The sludges recycled for biogas production were LSS, DS, and HSS and their surface and bottom layers. In E1, biogas yields from the recycled LSS, DS, and HSS did not differ (MW, *p* ≥ 0.453; Fig. 2). The compositions of the sludges were quite similar, the FS content ranging from 136 to 257 g/kg dry wt and the VS content ranging between 115 and 216 g/kg dry wt. In E2, by contrast, biogas yield from recycled DS (FS, 300 g/kg dry wt) was higher than from recycled HSS (FS, 375 g/kg dry wt; MW, *p* = 0.002), while that from LSS (FS, 83 g/kg dry wt) did not differ significantly from DS and HSS, due to large difference in biogas production between untreated (no biogas produced) and pH 7.0–adjusted LSS (MW, *p* = 1.000; Fig. 3, black bars of LSS, DS, and HSS). VS content was highest in DS (383 g/kg dry wt), moderate in HSS (308 g/kg dry wt), and lowest in LSS (100 g/g dry wt), and still, the biogas yield was highest in pH-adjusted LSS. VS adsorb the elements (Wong et al. 2002); i.e., the highest VS content of DS may have reduced the inhibitory effects of the elements by adsorbing and, at the same time, the highest VS and total C contents of DS supported biogas production (Fig. 3b; Table 2).

The biogas yields from the LSS and DS bottom layers were higher than from the surface layers (MW, *p* ≤ 0.044), although the VS contents were slightly higher than the FS contents in all layers (Fig. 3a, b). The anaerobic biogas-producing microbial community apparently has grown in the bottom layers during layer separation, while complex organic material may have risen to the surface where aerobic microorganisms also grow. Thus, the most easily accessible substrates for biogas production may have been at the bottom. Interestingly, the biogas production from DS surface and bottom layers was higher than from the original DS (MW, *p* ≤ 0.006), even though the layer materials were incubated for 28–33 days during surface and bottom separation by post-produced biogas. During the layer separation, some of the substances may disappear in the form of evaporated gases and some may dissolve and disappear with the added water, though generally, the elements were concentrated in the surface and bottom layers (Table 2). FS contents in biogas production from recycled SDS and BDS were lower than in DS experiments, although the FS percentages in DS (56.1%) and SDS/BDS (55.1%) were nearly the same (Fig. 3b). Dilution of FS and VS could increase biogas production in SDS/BDS compared with DS; ammonia dilution has also been found to increase biogas production (Chen et al. 2008).

In contrast to the LSS and DS, biogas production from the recycled surface layer of HSS was better than from the bottom layer, or from HSS (MW, *p* < 0.001), but the biogas production between recycled HSS and BHSS did not differ significantly (MW, *p* = 0.106; Fig. 3c). These differences were most likely due to the highest FS contents in HSS (375 g/kg dry wt) and BHSS (420 g/kg dry wt), while it was only 180 g/kg dry wt in SHSS. When the separation of surface and bottom layers increases the VS concentration on the surface, the biogas production from the surface VS can also be improved due to the decrease in FS.

Biogas production from digested sludges was generally low, and in some experiments, a reliable separation of methane and carbon dioxide would not have been possible due to the small volume; i.e., statistical analyses would not have been possible to do for methane volumes. Furthermore, the number of triplicate samples (required for statistical analyses) was 57 in E1, and 114 in E2, and performing a similar series of experiments on a larger scale would have been challenging. Biogas yields were low because easily available nutrients of the digested sludges had already been used for biogas production; E1 sludges had been shortly aerated; and all sludges were frozen prior to experiments due to the waiting time to separate layers, and even surface and bottom layers were frozen to ensure the same freeze-thaw treatments for all samples. For these reasons, methane was not separated from the biogas in this study. However, it is important to recall that all sludges were from full-scale biogas production and had the microbial community needed for anaerobic digestion. The proportion of methane in biogas was 56–58% at the BP, and slightly higher 60% at the WWTP, the LSS sludge of which was used as a microbial inoculum in this study. E3 showed that under saturated conditions, the bacteria in the sludge did not begin to consume ammonia and carbon for aerobic growth with increasing CO_2_ release, but apparently continued to produce biogas; the phenomenon was even utilized in separating surface and bottom layers. Moreover, despite the low volumes of biogas, many of the differences between the treatments were in good agreement with previous results in the literature, as discussed above. Although the volumes of produced biogas differed between E1 and E2, the differences between the same treatments were the same in the two experiments and supported the results of each other. In the following, the results are summarized based on E2.

#### Summary of treatments

**Fig. 5:**
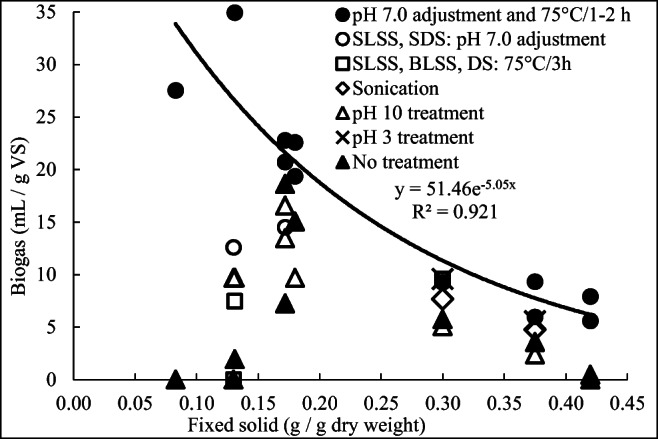
Biogas yields in different treatments of E2 as a function of the fixed solid content (the average of standard deviations, 3.3 mL/g VS; *n* = 3)

To evaluate the significance of the results, the biogas yields after the recycled sludge treatments in E2 were plotted as a function of FS (Fig. 5). The biogas yields after the best treatments (KW-MW, *p* < 0.050), that is after pH 7.0 adjustment and heating at 75 °C for 1–2 h, decreased exponentially with increasing amounts of FS according to the equation *y* = 51.46e^−5.05*x*^ (*R*^2^ = 0.921). The biogas yields after the other recycled sludge treatments were below this curve that is the FS content of the sludges did not limit the biogas production. Thus, heating for 3 h at 75 °C apparently had adverse effects on VS composition compared with heating for 1–2 h. For example, volatile fatty acids are formed increasingly in sludge under alkaline conditions (Chen et al. 2007), and they may have been evaporated during prolonged treatment, resulting in reduced biogas production. Sonication for 1 h may have had similar effects on VS, in agreement with the results in Segura et al. (2016). The inorganic ions added in the pH 3.0 (HCl) and 10.0 (KOH) treatments and neutralization increased the FS content of digested sludges, thereby enhancing the inhibition of biogas production by FS more than a simple pH adjustment. The inhibitory effect of increasing FS content appeared to be the major factor limiting biogas production.

Element additions (Fe, Ni, Co) have reduced biogas production from food waste with 90.8% of VS, and it has been explained to be due to the co-precipitation of trace elements essential for biogas production (Yazdanpanah et al. 2018). However, when Fe has been amended to the digestion of wastewater activated sludge, biogas production has been explained to be improved due to increased sludge VS oxidation (zero-valent iron and activated carbon; photo-Fenton pre-treatment; K_2_FeO_4_) (Heng et al. 2017; Wang et al. 2017). Combining these previous results with the results of this study, it can be estimated that, in addition to oxidizing the slurry VS, iron may have been involved in precipitating the FS, thereby reducing their inhibitory effects on biogas production, and resulting in improved VS digestion and biogas production. The FS content in wastewater sludge typically is higher than in food waste, about 33–38% (Feki et al. 2015; Heng et al. 2017; Liu et al. 2018). When the elements are added as part of the sludge pre-treatment prior to anaerobic digestion, the biogas-producing bacterial community may also have become adapted to higher elemental concentrations than the bacterial community of the untreated sludge, such as in anaerobic digestion of rice straw (Xin et al. 2019). Such a change in microbial community structure may enhance sludge VS anaerobic digestion. Nevertheless, the inhibitory effects of increasing FS content during digestion may be the most important factor that ultimately limits biogas production.

In general, microbiological hydrolysis or solubilization of complex carbon compounds like lignocellulose (cellulose, hemicellulose, lignin) has been considered as a rate-limiting step in anaerobic digestion (Chandra et al. 2012; Weiland 2010; Chiu and Lo 2016). However, in this study, biogas production was the highest in the recycled BLSS with the low FS content of 131 g/kg dry wt, although BLSS had already lost some of the readily available carbon in the separation of the surface and bottom layers of LSS by the biogas post-production for 33 days (Fig. 3). Correspondingly, the biogas yield from the heat-treated (75 °C, 2 h) and recycled SDS was higher than that from the recycled DS, which had a higher FS content (300 g/kg dry wt) than the SDS (172 g/kg dry wt) that had already undergone the separation of the surface and bottom layers with the loss of biogas. The digested sludge most likely contains microorganisms that degrade compounds like lignocellulose (Chandra et al. 2012; Goswami et al. 2016; Weiland 2010). The inhibitory effect of FS appeared to restrict biogas production from digested and recycled sludge more than the availability of carbon nutrients.

The percentage of VS did not fall below 38.5–56.1% at the end of biogas production (Figs. 2 and 3), which is in agreement with the earlier results (e.g., Zhang et al. 2008; Grübel and Suschka 2015; Liu et al. 2018). According to European Union legislation, the VS content in landfill must be less than 10% (European Council 1999), which prevents the digestate from being dumped in a landfill. The digested sludge can contain harmful compounds and drugs that inhibit the agricultural use of the sludge. The FS and water concentrations in the digested sludge were too high for profitable energy production, as most of the energy in the VS would be spent on evaporating the water. The inhibitory effects of inorganic ions should be circumvented, to further convert sludge VS into biogas. One possible method of concentrating 51.6–61.8% of VS on the surface is flotation, which could be further improved by adjusting the parameters.

## Conclusions

To improve digested sludge VS consumption, the VS of LSS, DS, and HSS were separated to surface and bottom layers using flotation by post-produced biogas, followed by adjusting the pH of the sludges to the optimum pH 7.0 for biogas-producing microbes; VS digestion with acid, alkali, heat, or sonolytic treatment; and finally biogas production measurements from recycled sludges. The biogas yield was best after the sole pH 7.0 adjustment, and after ammonia evaporation and VS digestion by heat treatment at 75 °C until the pH dropped to 7.0 (1–2 h), sonolytic treatment being almost as effective. Acid (HCl) and alkali (KOH) treatments were less effective in improving biogas production from the recycled digestates, as elevated FS levels appeared to limit biogas production more than difficult-to-digest VS.

To improve sludge VS digestion down to the European landfill limit of 10%, methods to concentrate VS and reduce FS should be found. In addition to FS dilution to avoid inhibitory effects, one possible method could be to separate VS into the surface and bottom layers by flotation using biogas post-production; 51.6–61.8% VS concentration was achieved in the surface layer using the non-optimized system. As VS and FS concentrations and compositions differ in surface and bottom layers, further optimization of conditions could improve VS utilization from both layers. When the sludge was stored for 35 days under aerobic conditions at 21 ± 2 °C, the pH fell most likely due to the use of ammonia as a substrate for microbial growth, whereas under saturated conditions, biogas production appeared to continue with ammonia and biogas evaporating without a decrease in pH. Thus, pH control and heat treatment to further digest sludge VS and adjust pH by removing ammonia (stripping) were the simplest methods for improving biogas production from the recycled sludge, while the biogas post-production at 21 ± 2 °C could be used to separate surface and bottom layers which differ in composition and digestibility.

## Abbreviations

BDS: Bottom layer of DS
BHSS: Bottom layer of HSS
BLSS: Bottom layer of LSS
BP: Biogas plant
DS: Dewatered sludge
E1: Experiment 1
E2: Experiment 2
FS: Fixed solids
HSS: High solid sludge
MW: Mann-Whitney test
KW: Kruskal-Wallis test
LSS: Low solid sludge
PCA: Principal component analysis
RMA: Repeated measures ANOVA
SDS: Surface layer of DS
SHSS: Surface layer of HSS
SLSS: Surface layer of LSS
VS: Volatile solids
WWTP: Wastewater treatment plant
